# The data set on vertical distribution pattern of *Bemisia tabaci* genn. (Homoptera: Aleyrodidae) in several vegetable crops

**DOI:** 10.1016/j.dib.2020.106157

**Published:** 2020-08-08

**Authors:** Sudarjat, Zesy Seftira, Luciana Djaya

**Affiliations:** Department Plant Protection, Faculty of Agriculture, Universitas Padjadjaran, Sumedang, 45363 Indonesia

**Keywords:** Distribution, Pest, Tropics, Vegetables

## Abstract

*Bemisia tabaci* is one of the main pests of vegetable crops in the tropics, including in Indonesia. The vertical distribution pattern of *B. tabaci* on investigated vegetable plants can be used to establish a sample observation unit in monitoring activities of pest control management. This data showed the vertical distribution pattern of *B. tabaci* on vegetable plants gathered from two different locations, namely from the high land of Ciwidey District, Bandung City (string bean, kidney bean, and tomato) and the low land of Sliyeg District, Indramayu City (eggplant, *towel gourd*, cucumber, and long beans). The data of *B. tabaci* nymph population were then analyzed to determine vertical distribution patterns.

**Specifications Table**

**Table:** 

Subject	Agricultural and Biological Sciences, Insect Science
Specific subject area	Agricultural and biological science, and focused on insect science. Insects are among the most important organisms affecting plant growth and development, with direct implications for crop quality and yield.
Type of data	Data tables.
How data were acquired	Data was acquired by observing the *Bemisia Tabaci* nymph population on vegetable plants originated from high and low land areas. Data were taken from 25 diagonal plant samples. In each planting area, data were taken from five plots, and in each plot, five plants were chosen randomly. Three-hundred leaf samples were taken per vegetable type: namely 100 young, 100 middle, and 100 old leaves.
Data format	Raw and Analyzed.
Parameters for data collection	Data were collected on the number of nymphs of *B. tabaci* in young, middle, and old leaves from seven vegetable crops. This data was used to calculate the distribution pattern, dispersion index value, χ2 value, and grouping index.
Description of data collection	The obtained data were analyzed using vertical distribution pattern analysis, one-way ANOVA, and Duncan's multiple range test (DMRT) at 5%.
Data source location	Institution: Universitas Padjadjaran City/Region: 1. Indramayu City/Sliyeg district, with the latitude of −6.414751 and 108.346069 2. Bandung City/Ciwidey district, with the latitude of **−***7.083062 and longitude of 107.447975* Country: Indonesia
Data accessibility	Within the article and on Mendeley Data with the DOI: doi: 10.17632/84xd3r3tg4.1 Direct URL to data: https://data.mendeley.com/datasets/84xd3r3tg4/1

**Value of the data**

• The data obtained here contribute to our understanding of the vertical distribution pattern of the *Bemisia tabaci* population on lowland and highland vegetable crops.
• These data will likely be useful for practitioners and farmers when planning pest control measures.
• The data can be used as basic data for reference in determining the best plant sample unit (leaf position) for the future experiment

## 1 Data description

The data report the vertical distribution patterns of *Bemisia tabaci* in seven vegetable crops originating from two locations: eggplant, towel gourd, cucumber, and long beans from high land of Ciwidey; and beans, kidney beans, and tomatoes from the low land of Silyeg. The vertical distribution pattern data of *B. tabaci* is important to determine on which leaves (young, middle, or old) the nymphs are clumped. This information can be used as a basis for taking leaf samples, where the nymphs cluster for integrated pest control. Several parameters related to the distribution of *B. tabaci* in various vegetable crops were analyzed. Data on the distribution pattern, dispersion index value, and χ2 value of seven vegetable crops are presented in Table 1. Table 1 also shows the suitability of the distribution patterns test of the seven vegetable crops.

**Table 1:** Distribution pattern, dispersion index value, and X^2^ value of seven vegetable crops.

Plant Species	Plant ages (days after planting)	Part Leaves Position	Distribution pattern	Dispersion Index	Chi Square (χ2)	χ2 (0,05)	χ2 (0,95)
Eggplant	30	Young	–	–	–	–	–
		Middle	Random	51.87	52.86	90.53	51.74
		Old	Clumped	199.79	141.08	101.88	60.39
String Beans	40	Young	Uniform	11.92	11.34	23.68	6.57
		Middle	Clumped	191.85	109.63	55.76	26.51
		Old	Clumped	220.69	155.43	79.08	43.19
Kidney beans	40	Young	Uniform	14.40	4.43	22.36	5.89
		Middle	Clumped	475.64	228.36	43.19	79.08
		Old	Clumped	130.20	148.44	43.19	79.08
Long beans	30	Young	Random	3.57	9.93	16.92	3.33
		Middle	Random	2.68	13.17	22.36	5.89
		Old	Random	2.21	9.21	2.17	16.92
Tomato	40	Young	Uniform	1.03	0.00	11.07	1.15
		Middle	Uniform	1.82	2.19	11.07	1.15
		Old	Clumped	1.46	59.53	11.07	1.15
Towel gourd	40	Young	Clumped	7.74	37.82	12.59	1.64
		Middle	Uniform	542.30	31.33	79.08	43.19
		Old	Clumped	199.14	2624.36	51.74	90.53
Cucumber	40	Young	Uniform	4.45	1.15	11.07	1.15
		Middle	Clumped	72.61	86.04	15.38	38.89
		Old	Uniform	54.23	32.62	67.50	34.76

On the young leaves, the population of *B. tabaci* nymphs was evenly distributed (follows a positive binomial distribution), while *B. tabaci* nymphs were clumped on the towel gourd. On the middle leaves, the population of *B. tabaci* nymphs was random, clumped, and uniformly distributed. On the old leaves, the population of *B. tabaci* nymphs was mostly clumped or follow a negative binomial distribution. The distribution pattern of the *B. tabaci* nymph population was obtained from the dispersion index value and χ2 value. The calculated χ2 values were compared with χ2 tables at = 0.05 and 0.95 with *n* = 1 degrees of freedom. χ2 tables (0.95, df = *N*-1) < χ2 counts < χ2 tables (0.05, df = *N*-1) show a random distribution, χ2 counts < χ2 tables (0.95, df = *N*-1) shows the distribution was evenly distributed, and when χ2 count > χ2 table (0.05, df = *N*-1) the distribution was clumped. String beans and kidney beans were two commodities with high population density, but the nymph populations on shoots do not form aggregates, where the distribution followed the positive or Poisson distributed binomials. As for the middle and old leaves, the population spreads following a negative binomial distribution.

Data on the grouping index value of *B. tabaci* nymphs for the seven vegetable crops are presented in Table 2. The grouping index values are influenced by the population mean values and their variance values. The smallest grouping index value shows the strongest grouping: the smallest k value of the seven vegetable plants was found from the young plant with a k value of 0.0632.

**Table 2:** Grouping index value of *B. tabaci* nymphs in several vegetable plants.

Plant Species	Part Leaves Position	Grouping Index (k)
Eggplants	Young	–
	Middle	0.5363
	Old	1.2143
Cucumber	Young	0.1678
	Middle	1.4109
	Old	0.6931
Towel gourd	Young	0.0632
	Middle	0.5692
	Old	0.6762
Long Beans	Young	0.4172
	Middle	3.7100
	Old	1.1168
Kidney Beans	Young	0.2710
	Middle	1.0107
	Old	1.2580
String Beans	Young	0.1929
	Middle	0.7173
	Old	1.1224
Tomato	Young	3.7800
	Middle	0.6539
	Old	0.2004

The average vertical distribution data for *B. tabaci* nymphs on vegetable plants from Ciwidey and Indramayu is presented in Table 3. Based on our observations, the nymphs were mainly found on the middle leaves of the seven vegetable crops from the Indramayu and Ciwidey areas, followed by the bottom leaves. In eggplant plants, string beans, kidney beans, towel gourd, and cucumbers, there were fewer *B. tabaci* nymphs on the young leaves than on middle and old leaves. Whereas in long bean and tomato, the number of *B. tabaci* nymphs in young, middle, and old leaves was not significant.

**Table 3:** Average of vertical distribution of *B. tabaci* nymphs on vegetable plants.

Part Leaves	Average of vertical distribution of *B. tabaci* nymphs						
	Eggplants	String Beans	Kidney Beans	Long Beans	Tomato	Towel Gourd	Cucumber
Young	0 a	2.31 a	2.54 a	1.78 a	0.13 a	0.51 a	0.38 a
Middle	48.89 b	35.51 b	95.87 b	3.39 a	0.65 a	79.17 b	8.47 b
Old	78.84 b	66.80 b	49.09 b	1.42 a	0.47 a	78.25 b	25.67 b

Note: The number followed by the same letter in the same column is not significantly different at the 5% level according to Duncan's multiple range test.

## 2 Experimental design, materials, and methods

### 2.1 Leaf sampling

Leaf samples were derived from plants that were attacked by *B. tabaci*, specifically eggplants, cucumbers, towel gourd, and long beans from the Sliyeg Subdistrict, Indramayu District, and kidney beans, string beans, and tomatoes from Ciwidey Subdistrict, Bandung District. Each plant was divided into three parts: young leaves, middle leaves, and old leaves. Four leaves were then taken per section. Samples were determined diagonally (consisting of five plots), plucked, put into plastic containers or jars that perforated for ventilation, and the total number of *B. tabaci* nymphs counted.

### 2.2 Observation of the *B. tabaci* nymph population

The number of *B. tabaci* nymphs was determined by counting all of the nymphs on a leaf sample. On each sample, all *B. tabaci* nymphs were counted.

### 2.3 Analysis of the distribution pattern, dispersion index, grouping index, and chi-square value

To determine the vertical distribution pattern of the population, the dispersion index (*I*) was calculated, and if the distribution was clustered, the grouping index (k) was also calculated. The vertical distribution pattern was tested using the Chi-square (χ2) test for goodness of fit [1]. To analyze the vertical distribution pattern, the following steps were applied:

Step 1: Calculating dispersion index and determining distribution pattern

The dispersion index was obtained from the following equation:

$$I=\;\frac{s^{2}}{\bar{x}}=\;\frac{\sum {\left(x_{1}-\;\bar{x}\right)}^{2}}{\bar{x}\left(n-1\right)}$$

Note: s^2^ = sample variant

$\bar{x}$ = average population density values

*n* = number of samples

*xi* = value x from 1,2,3,..etc.

Mean value from the sample:

$$\bar{x}=\frac{n}{N}$$

Variant value:

$$s^{2\;}=\;\left\{\left(\sum_{x=10}^{10}\left(x^{2}F_{x}\right)-\;\bar{x\;}n\;\right)/\left(N-1\right)\right\}$$

The distribution pattern can be determined from the value of *I* (i.e., *I* > 1 shows a clumped distribution pattern (negative binomial), *I* = 1 shows a random distribution pattern (Poisson), and *I* < 1 shows a uniform distribution pattern (positive binomial).

Step 2: The frequency distribution *Fx*

The frequency distribution was summarized as the number of individual nymphs per sample unit, that is the number of sample unit with 0, 1, 2, …, *r* number of individual nymphs. The data are summarized in the following table.

**Table:** 

x (number of nymphs per sample)				
$F_{x}$ (number of the same amount)				

Step 3: The negative binomial probabilities *P*(x) grouping index (*k*)

Negative binomial probabilities *P*(x) were used to calculate the probability of finding x individual nymphs in a leaf sample unit, where *x* = 0, 1, 2, …., *r* individual nymph. *P*(x) was calculated using the following equation:

$$P\left(x\right)=\;{\left[\bar{x}/\left(\bar{x}+\hat{k}\right)\right]}^{x}\left[\left(\hat{k}+x-1\right)!/\left(x!\left(\hat{k}-1\right)!\right)\right]{\left[1+\left(\bar{x}/\hat{k}\right)\right]}^{-k}$$

If the result of *I* analysis from the distribution pattern suitability test is clumped (negative binomial), then proceed with calculating the value of the grouping index (*k*).

The k (rough) value is obtained from Eq. (1)

(1) $$\hat{k}=\;\frac{{\bar{x}}^{2}}{s^{2}-\;\bar{x}}$$

The rough k value is substituted into Eq. (2) to obtain the fine k value.

(2) $$\hat{k}\;\log10=\;\left[1+\;\frac{\bar{x}}{\hat{k}}\right]=\;\log\left[\frac{n}{fo}\right]$$

Note:

*n* = number of samples

*fo* = number of samples with zero individuals

$\bar{x}$ = average population value

$\hat{k}$ = rough k value

Step 4: The expected negative binomial frequencies, *E*_x_

*Ex* represents the expected frequencies of *x* = 0, 1, 2, …., r individual nymphs as the following equation:

Ex=(N)P(x)

Note:

*N* = number of samples

P(x) = chance

Step 5: Goodness-of-fit test statistic, χ2

The chi-square χ2 test was used to ascertain how well the observed frequencies (*F*x in step 2) compare to expected frequencies (*Ex* in step 4). The chi-square χ^2^ was calculated using the following equation:

$$X^{2}=\sum_{\left(x=0\right)}^{q}\left\lceil {\left(F_{x}-E_{x}\right)}^{2}/E_{x}\right\rceil$$

χ^2^ = Chi-Square

*F*x = Frequency Distribution

*E*x = Expected Frequencies

The calculated χ2 value was compared to the χ2 table at a 5% significant difference with *n* = 1 degree of freedom. If the calculated χ2 value was greater than the table value, then the distribution was considered clumped.

### 2.4 Statistical analysis of the effect of leaf age on the vertical distribution of *B. tabaci* nymphs

The numbers of *B. tabaci* nymph populations found on young, middle, and old leaves were analyzed by analysis of variance (ANOVA). Furthermore, to determine the age of leaves that have an effect, the ANOVA was supplemented with Duncan's multiple range test at 5% significance level.

## Declaration of Competing Interest

The authors declare that they have no known competing financial interests or personal relationships which have, or could be perceived to have, influenced the work reported in this article.

## Appendix Supplementary materials

Image, application 1
